# The effect of enrofloxacin on enteric *Escherichia coli*: Fitting a mathematical model to *in vivo* data

**DOI:** 10.1371/journal.pone.0228138

**Published:** 2020-01-31

**Authors:** Samantha Erwin, Derek M. Foster, Megan E. Jacob, Mark G. Papich, Cristina Lanzas

**Affiliations:** 1 Department of Population Health and Pathobiology, College of Veterinary Medicine, North Carolina State University, Raleigh, NC, United States of America; 2 Department of Molecular and Biomedical Sciences, College of Veterinary Medicine, North Carolina State University, Raleigh, NC, United States of America; 3 Biomedical Sciences, Engineering, and Computing Group, Oak Ridge National Laboratory, Oak Ridge, TN, United States of America; University of Illinois, UNITED STATES

## Abstract

Antimicrobial drugs administered systemically may cause the emergence and dissemination of antimicrobial resistance among enteric bacteria. To develop logical, research-based recommendations for food animal veterinarians, we must understand how to maximize antimicrobial drug efficacy while minimizing risk of antimicrobial resistance. Our objective is to evaluate the effect of two approved dosing regimens of enrofloxacin (a single high dose or three low doses) on *Escherichia coli* in cattle. We look specifically at bacteria above and below the epidemiological cutoff (ECOFF), above which the bacteria are likely to have an acquired or mutational resistance to enrofloxacin. We developed a differential equation model for the antimicrobial drug concentrations in plasma and colon, and bacteria populations in the feces. The model was fit to animal data of drug concentrations in the plasma and colon obtained using ultrafiltration probes. Fecal *E. coli* counts and minimum inhibitory concentrations were measured for the week after receiving the antimicrobial drug. We predict that the antimicrobial susceptibility of the bacteria above the ECOFF pre-treatment strongly affects the composition of the bacteria following treatment. Faster removal of the antimicrobial drugs from the colon throughout the study leads to improved clearance of bacteria above the ECOFF in the low dose regimen. If we assume a fitness cost is associated with bacteria above the ECOFF, the increased fitness costs leads to reduction of bacteria above the ECOFF in the low dose study. These results suggest the initial *E. coli* susceptibility is a strong indicator of how steers respond to antimicrobial drug treatment.

## Introduction

Fluoroquinolones are broad-spectrum antimicrobial drugs with concentration-dependent bactericidal activity [1]. They are categorized as critically important antimicrobial drugs for human medicine [2, 3]. An important public health concern with fluoroquinolones is their increased resistance in foodborne pathogens such as *Campylobacter* and *Salmonella* because fluoroquinolones are one of the treatment choices for complicated cases caused by both pathogens [4, 5]. Increased fluoroquinolones resistance in foodborne pathogens can be driven by the use of antimicrobial drugs in veterinary medicine because food animals are the main reservoir for these pathogens [6]. To minimize public health risk, the Food and Drug Administration (FDA) has prohibited the extra-label use of fluoroquinolones in food animals. Currently, two fluoroquinolones are approved in the United States for food animals: enrofloxacin and danofloxacin. These drugs are approved for bovine and swine respiratory diseases in a injectable form. As extra-label use is prohibited, the drugs cannot be used for other indications, dose, route of administration or species beyond that is indicated in the label.

Fluoroquinolones can reach high concentrations in the gut because they are partially excreted in the bile acid [7]. Additionally, intestinal efflux transporters may transport fluoroquinolones into the gut lumen [8]. As a result, fluoroquinolones can disrupt the gut commensal bacteria— even when treatment is parental [9]. In addition to influencing the population dynamics of enteric bacteria, fluoroquinolones may select for resistance. Resistance is most often mediated by mutations on the genes coding fluoroquinolone targets: DNA-gyrase and topoisomerases [10]. The increase in resistance during treatment is often attributed to the selection and amplification of pre-treatment mutants [11]. Resistance following oral treatment has been reported in broilers and swine [11–13]. On the contrary, intramuscular high doses in swine and cattle have been associated with little resistance, suggesting that route of administration and dose may influence fluoroquinolones’ effect on resistance in enteric bacteria [11, 14]. In veterinary medicine, dose regimes are approved based on efficacy [1]. Therefore, the pharmacokinetics-pharmacodynamics (PK-PD) of the drug influence on enteric bacterial populations, including resistance selection, is less understood.

To investigate what factors influence the effect of enrofloxacin on enterobacteriacea, we developed a mathematical model to represent the dynamics of enrofloxacin in steers following subcutaneous administration and its effects on *E. coli* population in the gut. The model was fit to data from twelve steers that were monitored following two dose regimens: a single high dose or three low doses [15]. These are currently the only two approved doses of fluoroquinolones in cattle.

## Materials and methods

### Experimental data

The experimental data have been previously described in [15] and was approved by the North Carolina State University Institutional Animal Care and Use Committee. Briefly, twelve healthy six-month-old steers were assigned to two dose regimes. The steers ranged in weight from 124-241 kg with similar body condition scores (2.5-3.5). The treatments were a single high subcutaneous dose of enrofloxacin (12.5 mg/kg) or lower subcutaneous doses of enrofloxacin (5.0 mg/kg), administered 24 hours apart for three doses. Ultrafiltration probes were placed in each steers’ ileum and spiral colon. Enrofloxacin is partially metabolized into ciprofloxacin [16]. Because enrofloxacin and ciprofloxacin produce additive antimicrobial activity, we combined both antimicrobial drugs and modeled the total concentration of both drugs. Following a subcutaneous administration, measurements of the enrofloxacin and ciprofloxacin concentrations were taken in the plasma, interstitial fluid, ileum, and colon. In addition to the antimicrobial drug concentrations, fecal samples were collected by hand and *E. coli* concentration was measured in colony forming units per milliliter (CFUs/ml) [15]. In the high dose treatment, fecal samples were taken every 12 hours for the first 48 hours, then 24 hours there after until hour 192. In the low dose treatment, fecal samples were taken every 12 hours for the first 96 hours, then 24 hours there after until hour 192. The minimal inhibitory concentration (MIC) of *E. coli* to enrofloxacin were measured for 8 isolates at several time points using the approved Clinical and Laboratory Standards Institute (CLSI) standard [17]. At the conclusion of the study and observation of the appropriate meat withdrawal time, all ultrafiltration probes and catheters were removed, and the steers were sold.

### Mathematical model

**Fig 1:**
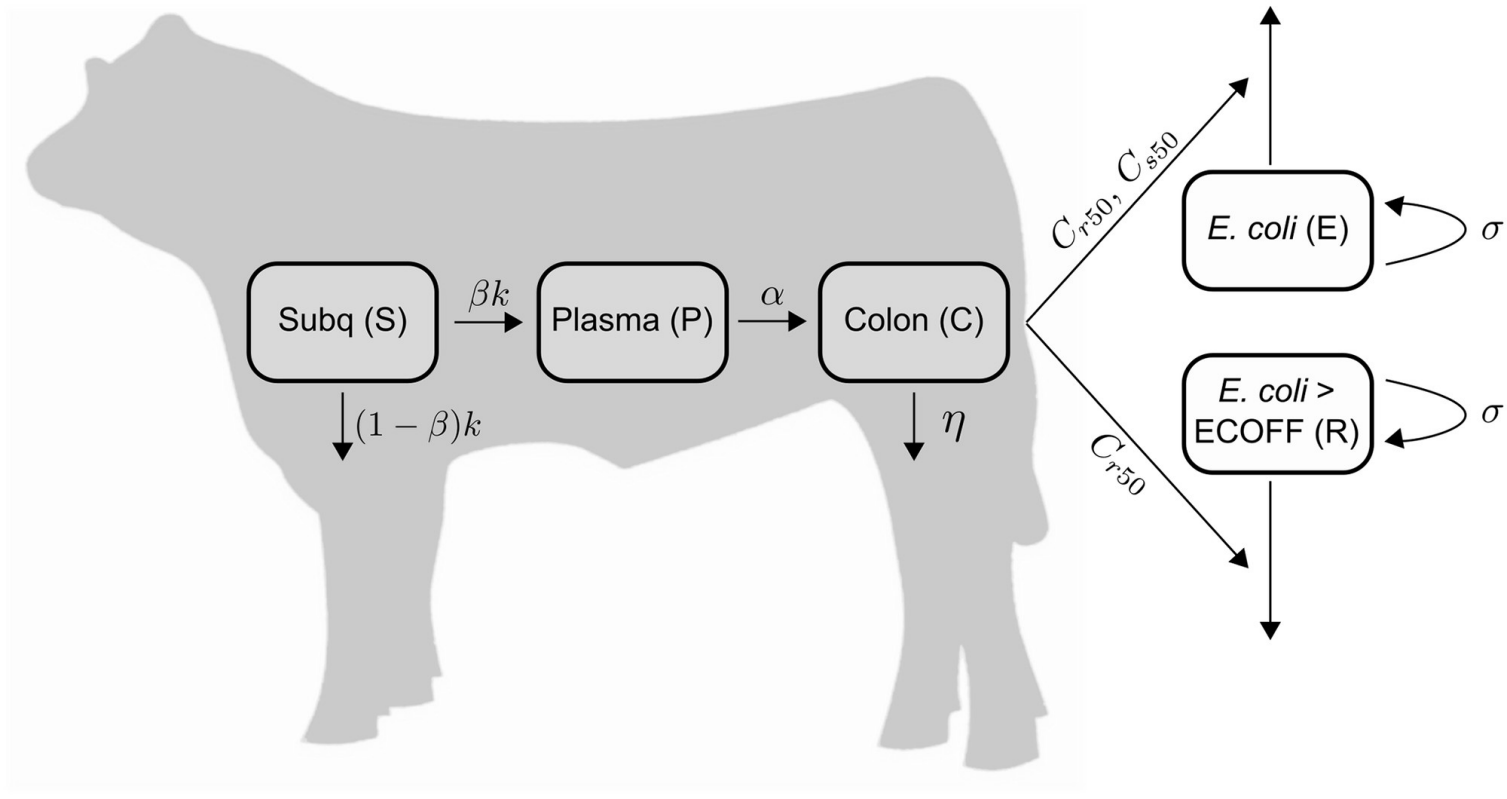
ODE schematic. Schematic of the mathematical model for enrofloxacin and ciprofloxacin concentrations throughout the gastrointestinal tract (GIT) of the steer and the effects on the *E. coli* population. The compartments S, P, and C are antimicrobial concentration in the steer while the compartments E and R describe the bacteria population in the feces.

An ordinary differential equation model was developed to describe the dynamics of the antimicrobial drug and bacterial population in the host. Fig 1 presents the model flowchart. The choice of the model structure was based on the ability to identify the model parameters with the available data. More complex models were not identifiable given the available data.

In our model, enrofloxacin is administered subcutaneously (*S*) with a dose *S*_0_ given *n* times with time intervals *T*, modeled by the Dirac function *δ*(*t* − *iT*). The subcutaneous compartment is modeled as a single compartment with linear elimination at rate *k*. The antimicrobial drug is then distributed into the plasma at rate *βk*; this term accounts for the amount of drug that reaches the plasma. *P* is the total concentration of bound and unbound antimicrobial drug in the plasma. From the plasma, the antimicrobial drugs are transported to the colon (*C*) at a net transfer rate *α* by means of passive diffusion, and active transport into the small intestine with subsequent transit to the colon. *C* is the total concentration of unbound drugs in the colon. Finally, the drugs are eliminated from the colon at rate *η*. We assume all rates are for enrofloxacin and its metabolized form ciprofloxacin combined.

We assume *E. coli* grows logistically at rate *σ*, and the maximum *E. coli* concentration is *N*_*max*_. In addition to modeling changes on the overall *E. coli* population (E), we follow the subpopulation with MIC above the epidemiological cut-off value (ECOFF), (R). Based on the European Committee on Antimicrobial Susceptibility Testing, the MIC epidemiological cut-off value for enrofloxacin on *E. coli* is 0.125 *μ*g/ml [18]. The pharmacodynamic effects of the antimicrobial drug on bacteria above (*R*) and below (*E* − *R*) this threshold is captured through *C*_*r*50_ and *C*_*s*50_, respectively. These parameters represent the concentration of drug needed to produce 50% of the maximum killing effect. Ciprofloxacin is more active than enrofloxacin against gram-negative bacteria, thus our decision to combine both drugs introduces some bias on the effects on bacteria [19]. Our pharmacodynamic parameters (*C*_*r*50_ and *C*_*s*50_) may not be directly comparable to pharmacodynamic parameters obtained for each drug independently.

The system of equations for the model described above is given by:

$$\begin{array}{ccc} \frac{dS}{dt} & = & \sum_{i=0}^{n}S_{0}\delta \left(t-iT\right)-kS,\\ \frac{dP}{dt} & = & \beta kS-\alpha P,\\ \frac{dC}{dt} & = & \alpha P-\eta C,\\ \frac{dE}{dt} & = & \sigma \left(1-\frac{E}{N_{max}}\right)E-\frac{C}{C+C_{s50}}\left(E-R\right)-\frac{C}{C+C_{r50}}R,\\ \frac{dR}{dt} & = & \sigma \left(1-\frac{E}{N_{max}}\right)R-\frac{C}{C+C_{r50}}R, \end{array}$$ (1)

with initial conditions *S*(0) = 0 mg/kg, *P*(0) = 0 *μ*g/mL, *C*(0) = 0 *μ*g/mL, *E*(0) = *E*_0_ log10 CFUs and *R*(0) = *R*_0_ log10 CFUs.

The relation between mutations that confer fluoroquinolone resistance and bacteria fitness is complex [20]. Some resistance mutations that decrease susceptibility to fluoroquinolones decrease bacterial fitness. In other instances, accumulation of multiple mutations conferring resistance can lead to improved fitness [20]. To investigate the effect of fitness costs after treatment, we adjusted the bacterial growth to account for differential fitness. Our initial model assumes that all of the *E. coli* grow identically. Here we investigate fitness cost associated with the bacteria above the ECOFF. In the following model we denote *c* as the fitness cost.

$$\begin{array}{ccc} \frac{dS}{dt} & = & \sum_{i=0}^{n}S_{0}\delta \left(t-iT\right)-kS,\\ \frac{dP}{dt} & = & \beta kS-\alpha P,\\ \frac{dC}{dt} & = & \alpha P-\eta C,\\ \frac{dE}{dt} & = & \sigma \left(1-\frac{E}{N_{max}}\right)\left(E-cR\right)-\frac{C}{C+C_{s50}}\left(E-R\right)-\frac{C}{C+C_{r50}}\left(R\right),\\ \frac{dR}{dt} & = & \left(1-c\right)\sigma \left(1-\frac{E}{N_{max}}\right)R-\frac{C}{C+C_{r50}}R, \end{array}$$ (2)

with initial conditions *S*(0) = 0 mg/kg, *P*(0) = 0 *μ*g/mL, *C*(0) = 0 *μ*g/mL, *E*(0) = *E*_0_ log10 CFUs, *R*(0) = *R*_0_ log10 CFUs, and *S*_0_ = *D* mg/kg. We use this model to investigate the fitness cost associated with bacteria above the ECOFF.

### Identifiability, parameterization, and sensitivity analysis

Parameters that can be uniquely derived from data are identifiable. We investigated the structural identifiability of the model prior to performing parameter estimation. For our model, all of the parameters are unknown except *S*_0_ and *k*. Using the web application COMBOS [21], we confirmed that *β*, *α*, *η*, *σ*, *N*_*max*_, *C*_*s*50_ and *C*_*r*50_ were uniquely identifiable parameters. The exact code used in the web application is in the appendix.

The software Monolix 2018 R1 (Lixoft, Antony, France) was used to perform all parameter estimations. Using our model (1), we fit the model to the data for each steer in the high and low dose treatment group independently. The parameters in the model were estimated using stochastic approximation expectation maximization (SAEM) over a maximum of 200 iterations with a Monte Carlo sampling size of 10, 000. SAEM combines a maximum likelihood estimation algorithm with a stochastic approximation procedure for estimating conditional expectations [22]. We fixed *k* based on previous studies [14], see Table 1. We assumed all parameters had log-normal distributions. Furthermore, we assumed that the drug parameters could vary across the two cohorts as it has been previously shown that the transport mechanism in the colon may be concentration dependent [15]. Similarly, we let *N*_*max*_ vary and *σ* vary across study groups to capture *E. coli* dynamics at the individual level. Commensal *E.coli* population vary across individuals and can be highly dynamic [23]. We estimated *β*, *α*, *η*, *σ*, *N*_*max*_, *C*_*r*50_, *C*_*s*50_ and *r*_0_ for the low dose study. Due to the lack of *E. coli* above the ECOFF during the high dose study, it was not possible to fit *C*_*r*50_ in the high dose study. Thus we used the *C*_*r*50_ fit in the low dose study and fixed it for the high dose study.

**Table 1:** Parameter estimates.

Parameter	Description	Units	HD *μ*	HD *ω*	LD *μ*	LD *ω*
*k*	drug absorption rate into plasma	$\frac{1}{\text{hr}}$	0.44	fixed	0.44	fixed
*β*	drug conversion factor into plasma	$\frac{\mu \text{g}\;\text{kg}}{\text{mg}\;\text{ml}}$	0.00127	0.31	0.00157	0.339
*α*	net drug transfer rate into colon	$\frac{1}{\text{hr}}$	0.163	0.316	0.146	0.192
*η*	elimination of drug from colon	$\frac{1}{\text{hr}}$	0.0464	0.605	0.0854	0.0275
*σ*	*E. coli growth rate*	$\frac{1}{\text{hr}}$	0.211	0.371	0.188	0.0349
*N*_*max*_	maximum bacteria concentration	$\frac{\text{logCFUs}}{\text{g}}$	5.52	0.044	6.21	0.077
*C*_*s*50_	50% of sensitive bacteria killing effect	$\frac{\mu \text{g}}{\text{mL}}$	6.05	0.546	2.02	0.689
*C*_*r*50_	50% of bacteria above ECOFF bacteria killing effect	$\frac{\mu \text{g}}{\text{mL}}$	5.91	fixed	5.91	0.339
*r*_0_	initial amount of bacteria above ECOFF	$\frac{\text{logCFUs}}{\text{g}}$	5.74 × 10^−5^	7.27	1.39	0.696

Population parameter estimates of the parameters: *k*,drug conversion factor into plasma; *β*, drug distribution into plasma; *α*, net drug transfer rate into colon; *η*, elimination of drug from colon; *σ*, *E. coli* growth rate; *N*_*max*_, maximum bacteria concentration; *C*_*s*50_, 50% of sensitive bacteria killing effect; *C*_*r*50_, 50% of bacteria above the ECOFF killing effect; and *r*_0_, initial amount of bacteria above the ECOFF. The median of distribution of the parameter estimate for the high dose and low dose steers in the columns, HD *μ* and LD *μ*, respectively. The non-transformed lognormal standard deviation of the high dose and low dose steers is also in the columns HD *ω* and LD *ω*. We fixed *k* based on previous studies [14], and *C*_*r*50_ in the high dose steers based on the low dose estimation.

In addition to fitting the individual-level parameters, we obtained population-level estimates. We estimated population-wide parameters using a two step process: (1) a Bayesian estimate of the individual parameters; and (2) parameters were estimated using a linearization about those Bayesian estimates [24]. The simulations output the transformed population parameters, *μ*, while the given standard deviation, *ω*, is untransformed. The estimated population distribution for each parameter is $\text{X}(\text{i})=\mu e^{\eta_{i}}$ where $\eta_{i}∼N(0,\omega^{2})$.

Using the R package Sensitivity [25], we also performed a sensitivity analysis of the model parameters under different dosing regimens. We chose a uniform distribution for the parameters, where the minimum and maximum are + /− 3 standard deviations from the population level mean. We compared the parameters’ effects on the total cumulative bacteria above the ECOFF at the end of the simulation (i.e we compared the amount of $\int_{0}^{t_{end}}Rdt$). We implemented the extended Fourier amplitude sensitivity test which estimates the first order and total Sobol indices for each parameter [26]. The first order effects measure the variation caused by the parameter alone; the total effects measure the overall effect of varying the parameter, including variance caused by parameter interactions.

## Results

### Data fitting

**Fig 2:**
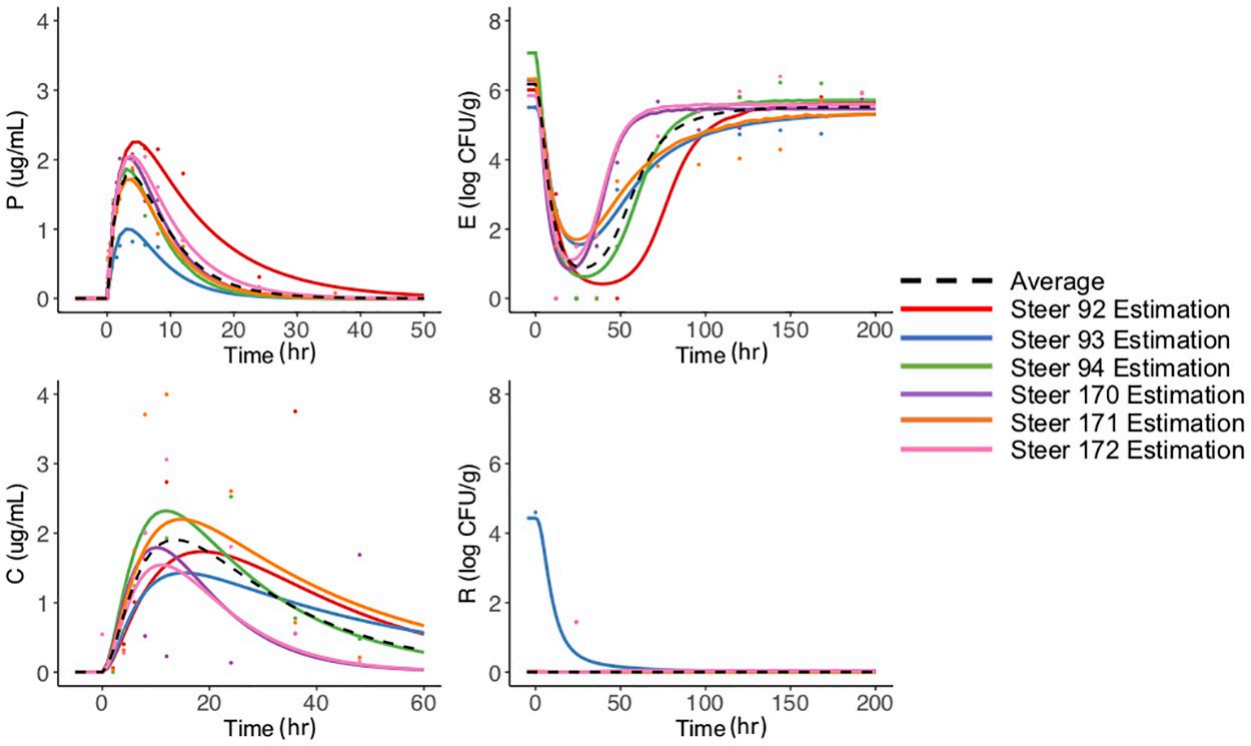
High dose steer dynamics. Prediction of the high dose treatment group. Estimation of the concentration of antimicrobial drugs in the plasma, *P*, colon, *C* and estimation of the amount of the total *E. coli* and *E. coli* above the ECOFF in the feces. Each steer is represented by the same color for model predictions (lines) and corresponding data (dots). The median population estimate for each compartment is the black dashed line.

**Fig 3:**
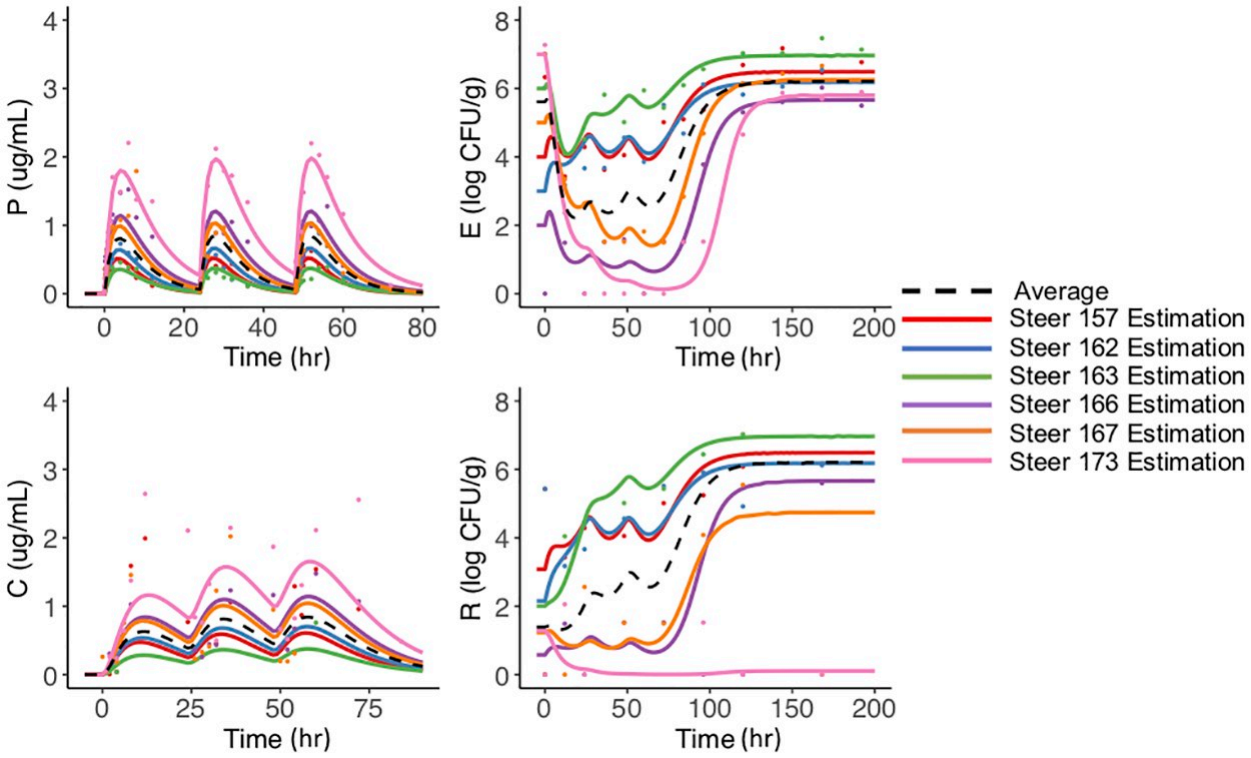
Low dose steer dynamics. Prediction of the low dose treatment group. Estimation of the concentration of antimicrobial drugs in the plasma, *P*, colon, *C* and estimation of the amount of the total *E. coli* and *E. coli* above the ECOFF in the feces. Each steer is represented by the same color for model predictions (lines) and corresponding data (dots). The median population estimate for each compartment is the black dashed line.

For each steer, we fit the model to the concentration of antimicrobial drug (enrofloxacin and ciprofloxacin together) in the plasma and colon, and we fit the *E. coli* population in the feces and the above the ECOFF subpopulation. In Figs 2 and 3, we plotted the individual steer data (circles) with the corresponding fit in the same line color for the high and low dose respectively. The summary of the individual parameters is provided in the appendix in S1 and S2 Tables. No steer previously received antibiotics however. It is possible they were housed with treated animals prior to their arrival, which may have contributed to the different initial amounts of R populations. At the high dose, *E. coli* population changes over time are fairly similar across individuals. All the animals had an overall decrease in the total *E. coli* population and a subsequent recovery within 100 hours post-dose (Fig 2). Most animals had no *E.coli* above the ECOFF at time zero, therefore the model did not predict further selection. In a following section, we further explore the effect of modifying the initial conditions. Although for the low dose, the pattern of trajectories for *E. coli* over time was similar across individuals, there was more variation on the nadir reached by the *E.coli* compared to the high dose (Fig 3). All of the steers had similar patterns for the percentage of bacteria above the ECOFF except steer 173, which had less *E. coli* above the ECOFF after treatment than before treatment. These differences are likely attributed to inter-animal variability, which is commonly seen in pharmacological studies.

We also estimated the population-wide parameters for both the high and low dose populations (Table 1). Population-level parameters were similar across treatments. The parameter *β* accounts for the distribution of antimicrobial drugs in the plasma and is a conversion from the mg/kg dose given to each steer to the *μ*g/ml drug concentration in the plasma and colon. This accounts for the lower measurement of *β*. *β* varied between the dosing regimens, with a change of 3 × 10^−4^ *μ*g/mgml, or a 24% increase from the high dose cohort to low dose cohort estimate. *β* varied between the dosing regimens, with the low-dose treatment having 3 × 10^−4^ *μ*g/mgml, or a 24% higher level, versus the high-dose treatment. The movement of the drugs into the colon via the plasma, rate *α*, decreased by 10% for the high-dose treatment as compared to low-dose population estimates. The elimination of antimicrobial drugs from the colon, *η*, in the high-dose regimen was nearly double that of the low dose regimen. The bacterial growth rate, *σ* decreased by 14% in the low dose regimen, which is an indication of the slower growth of *E. coli* after multiple rounds of antimicrobial drugs. There is a slight variation in *N*_*max*_ across the dosing regimens (11% change from high to low dose). Similarly, we see a large variation in *r*_0_ but this is expected given the variation in the initial amount of bacteria above the ECOFF between the high dose and low dose steers. Finally, *C*_*s*50_ has a significant variation across the cohorts, nearly three times larger in the high dose regimen. The steers are fit individually across the cohorts and this is only a comparison of the mean estimation. This is primarily an artifact of the small to non-existent amount of bacteria above the ECOFF, thus *C*_*s*50_ is fit to more of an overall *C*_50_ of antimicrobial drugs in the high dose steers.

**Fig 4:**
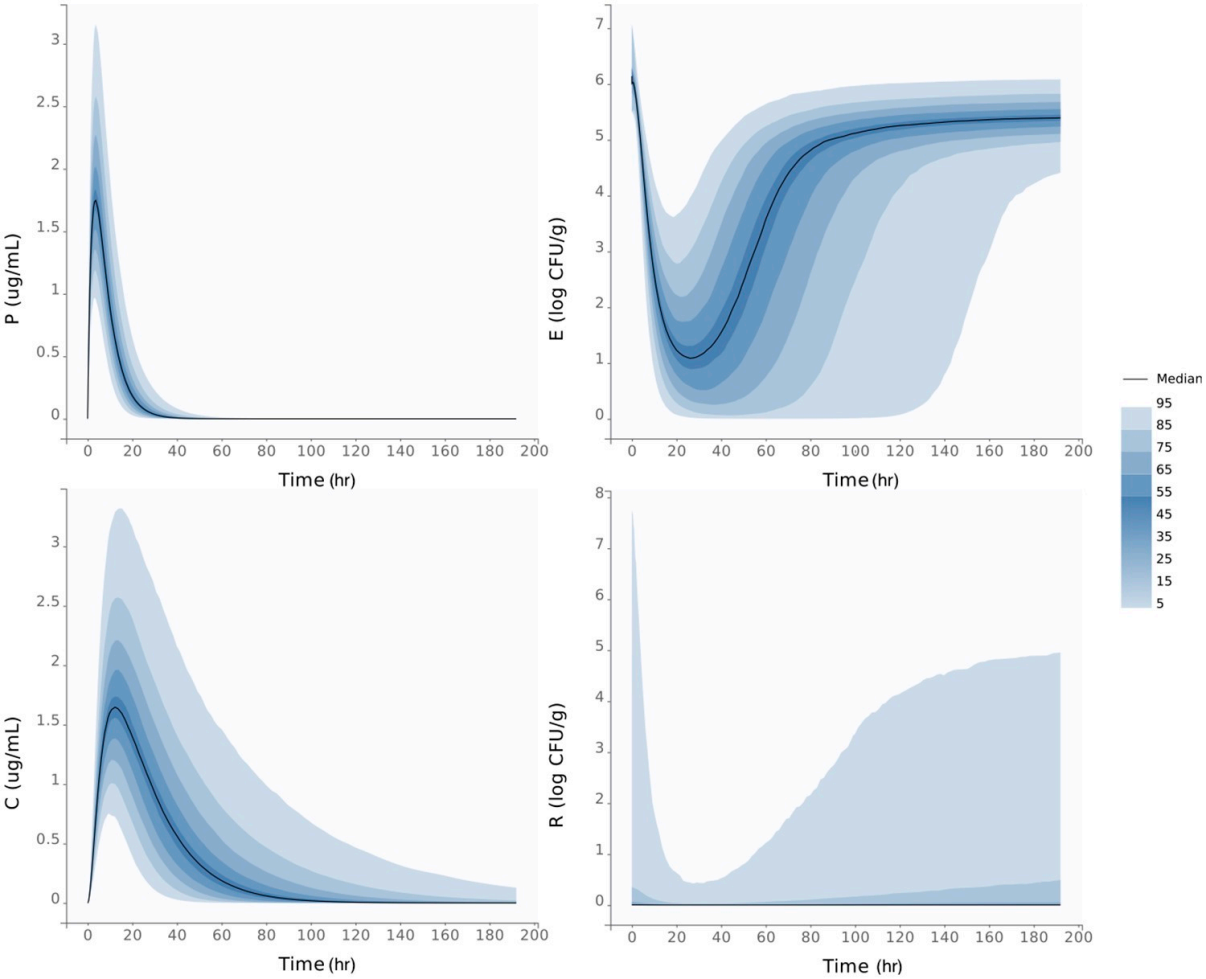
High dose distributions. Predicted distribution of our model for the antimicrobial drugs concentration throughout the GIT of the steer during the high dosing regimen. The top left panel is the plasma, the bottom left is the colon, top right the total *E. coli* population and bottom right is *E. coli* above the ECOFF. In each panel, the dark line represents the median, and the darkest blue region has a 50% likelihood, whereas the lighter colors are less likely to occur.

Based on the median and standard deviation for the parameters of both the high dose and low dose treatments, we plotted the predicted distribution of both dosing regime groups. In Fig 4, we plot the distribution of the plasma, colon, total *E. coli* and *E. coli* above the ECOFF during the high dosing regimen. The region of distribution is small for the plasma, with the smallest predicted peak of 1 *μ*g/mL and the maximum is 3 *μ*g/mL of antimicrobial drugs in the plasma, both predictions occur within the first 12 hours. Alternatively there is a larger variability in the colon ranging from the smallest predicted peak of 0.8 *μ*g/mL and the highest predicted peak of 3.5 *μ*g/mL of antimicrobial drugs in the colon, both of which occur within the first 24 hours. There is a 90% likelihood that the *E. coli* population returns to a steady state between 5 and 6 log CFU/g within 200 hours. Also noteworthy is the variability in the bacteria above the ECOFF population, which is likely due to the variability in *C*_*r*50_. There is a 95% likelihood the amount of bacteria above the ECOFF will stay below 1 log CFU/g.

**Fig 5:**
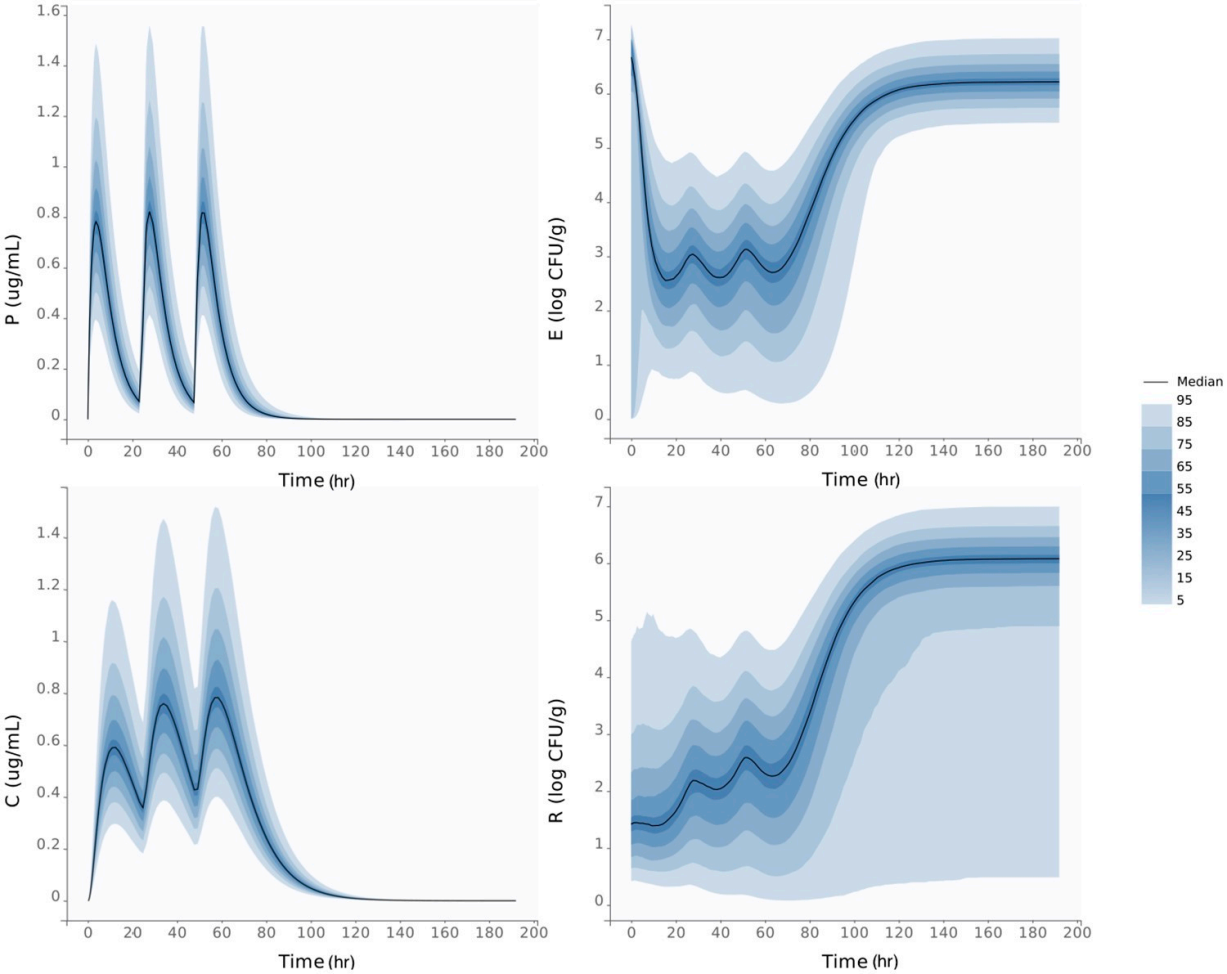
Low dose distributions. Predicted distribution of our model for the antimicrobial drug concentration throughout the GIT of the steer during the low dosing regimen. The top left panel is the plasma, the bottom left is the colon, top right is the total *E. coli* population and bottom right is the amount of *E. coli* above the ECOFF. In each panel, the dark line is the median, and the darkest blue region has a 50% likelihood, whereas the lighter colors are less likely to occur.

Similarly, in Fig 5, we plot the predicted population-level distribution of the plasma, colon, total *E. coli* and *E. coli* above the ECOFF during the low dosing regimen. The region of distribution is small for the plasma, with the smallest predicted peak at 0.4 *μ*g/mL and the maximum at 1.6 *μ*g/mL of antimicrobial drugs in the plasma. Alternatively there is larger variability in the colon ranging from the smallest predicted peak of 0.3 *μ*g/mL and the highest predicted peak of 1.6 *μ*g/mL of antimicrobial drugs in the colon. The *E. coli* population returns to a steady state ranging from 5 to 7 log CFU/g for all predictions within 140 hours for the low dosing regimen. Also noteworthy is the large variability in the amount of *E. coli* above the ECOFF. Our model can predict an *E. coli* population that is completely above the ECOFF, but may also predict as few as 1 CFU/g depending on the parameters for the low dosing regimen.

### Sensitivity analysis

We performed a sensitivity analysis of the parameters for the high and low dose models. In Table 2, we include the range for each parameter during the sensitivity analysis for the high and low dose regimen. We bound our parameters by +/-3 of the standard deviations. The maximum *r*_0_ is bound on the upper side, by the initial amount of *E. coli*. During our simulations, we compared how the parameter changes affected the area under the curve of the amount of bacteria above the ECOFF.

**Table 2:** Sensitivity analysis parameter ranges.

Parameters	Min High Dose	Max High Dose	Min Low Dose	Max Low Dose
*β*	0.00087	0.0017	0.0011	0.0022
*α*	0.0017	0.22	0.13	0.16
*η*	0.011	0.14	0.085	0.086
*σ*	0.12	0.32	0.19	0.19
*N*_*max*_	5.48	5.55	6.10	6.32
*C*_*s*50_	1.84	14.80	0.49	8.39
*C*_*r*50_	-	-	4.19	8.34
*r*_0_	0	5.6	0.33	6.16

Parameter values used to bound the global Sobol sensitivity analysis. The minimum and maximum values are given for each parameter for the high dose and low dose dosing regimens.

**Fig 6:**
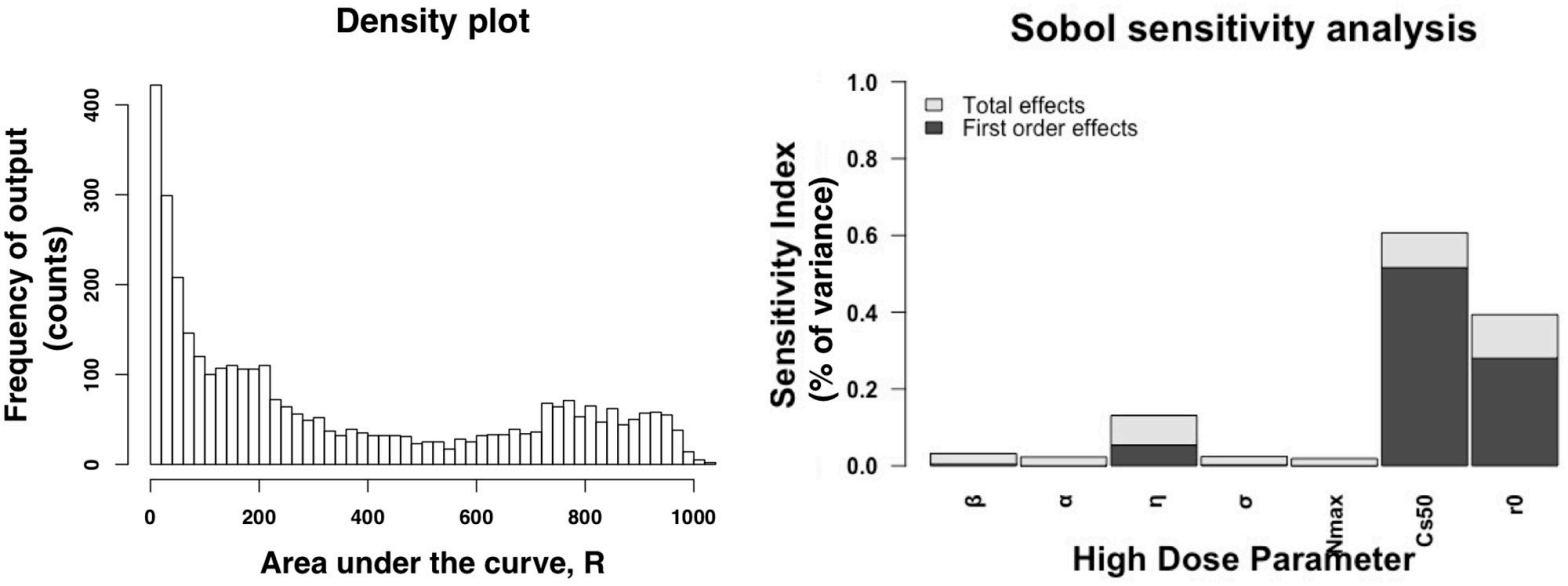
High dose sensitivity analysis. Sobol sensitivity analysis results of the high dose regime. The left panel is a histogram of the output variable of the sensitivity analysis, the area under the curve of the bacteria above the ECOFF, *R*. The right panel is the sensitivity index for each parameters. The first order effects measures varying the parameter alone but averaged across all of the other variations while the total effect measures the effect of varying the parameters including variance caused by the parameters interactions.

We simulate model outcomes for 3,500 simulations across the parameter space during the high dose regimen (500 variations of the 7 parameters). In the left panel of Fig 6, we plot the frequency of the outcome, the area under the curve of the amount of bacteria above the ECOFF. During the high dose simulations, the majority of the simulations have an area under the curve that is less than 100. Since our simulation is for over 200 hours, during the duration of the simulation the mean value of *R* is less than 0.5 log 10 CFUs/g. These results indicate that for most of the parameter space, the bacteria above the ECOFF is not dominant, and eventually dies out. We also plotted the first order effects and total effects on the total amount of bacteria above the ECOFF over time during the high dose simulation, in Fig 6 right panel. Based on our sensitivity analysis, we found that *C*_*s*50_, 50% of the bacteria killing effect is the most sensitive parameter. This is an expected outcome given that this parameter strongly controls the effect the antimicrobial drug has on the amount of bacteria. Additionally, the initial amount of bacteria above the ECOFF, *r*_0_, strongly effects the amount of overall amount of bacteria above the ECOFF. The parameter *η* has the strongest effect on the overall amount of bacteria above the ECOFF of all the drug parameters.

**Fig 7:**
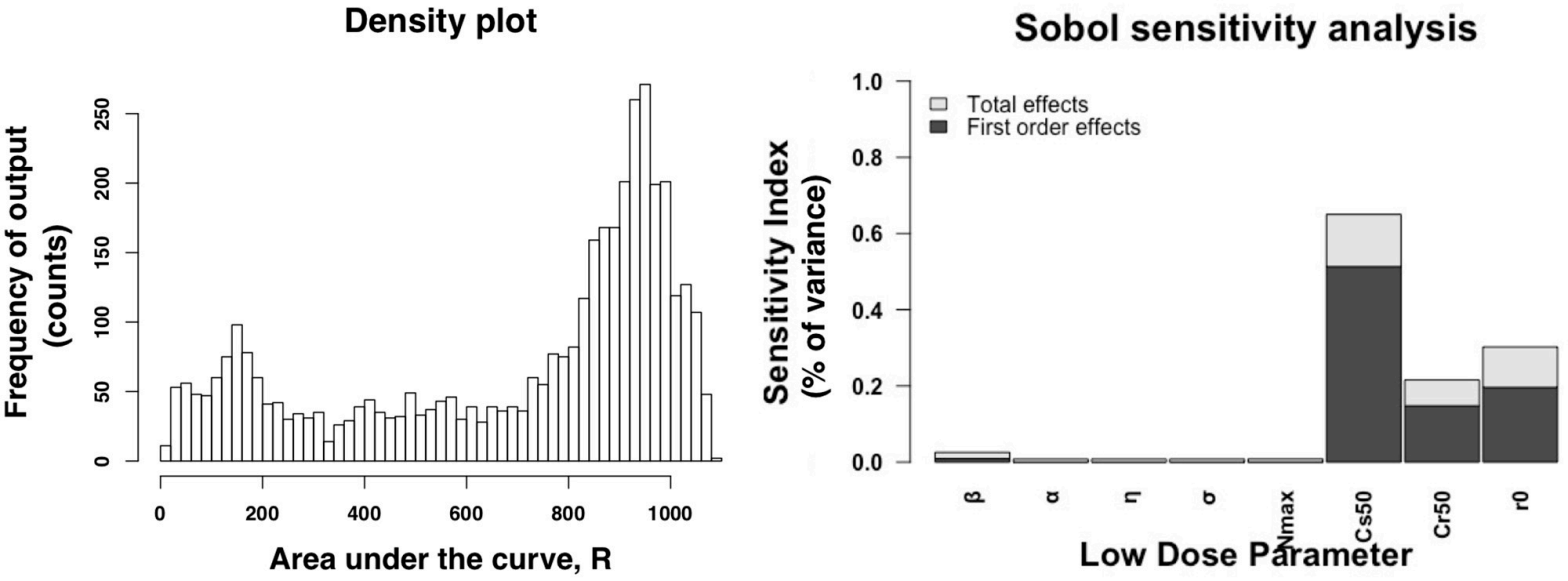
Low dose sensitivity analysis. Sobol sensitivity analysis results of the low dose regime. The left panel is a histogram of the output variable of the sensitivity analysis, the area under the curve of the bacteria above the ECOFF, *R*. The right panel is the sensitivity index for each parameters. The first order effects measures varying the parameter alone but averaged across all of the other variations while the total effect measures the effect of varying the parameters including variance caused by the parameters interactions.

We simulate model outcomes for 4,000 simulations across the parameter space during the low dose regimen (500 variations of the 8 parameters). In Fig 7, left panel, we plot the frequency of the outcome, the area under the curve of the bacteria above the ECOFF, of the sensitivity analysis. During the low dose simulations, the simulations range from 5 to 1,300 log 10 CFUs/g total amount of bacteria above the ECOFF over 200 hours. Most of the simulations range from 800 to 1,200 log 10 CFUs/g total amount of bacteria above the ECOFF over 200 hours. Since our simulations occur over 200 hours, the mean value for R is *R* = 5 log10CFUs/g. These results indicate that for most of the parameter space, the bacteria above the ECOFF dominate the population. For the low dose regimen, we also find C_*s*50_, the half maximum killing effect of the antimicrobial drug on the total bacteria is the most sensitivity parameter (Fig 7, right panel). The second most influential parameter is again *r*_0_, the initial amount of bacteria above the ECOFF. For the drug parameters, *β* is the most sensitive in the low dose regimen, this varies from the high dose regimen. The area in which were sampling *η* is small due to little variation in the estimation in the low dose steers. If a larger range is sampled, *η* is also sensitive in these simulations.

### Simulating the effects of individual parameter changes

Based on our results in the predicted distribution and the sensitivity analysis, there is little variation in the total amount of bacteria above the ECOFF in the high dosing regimen. We focus on understanding ways we can decrease or increase the amount of bacteria above the ECOFF developed during antimicrobial drug treatment by individually modifying the parameters beyond the range in the sensitivity analysis.

#### Variations in *C*_*s*50_

**Fig 8:**
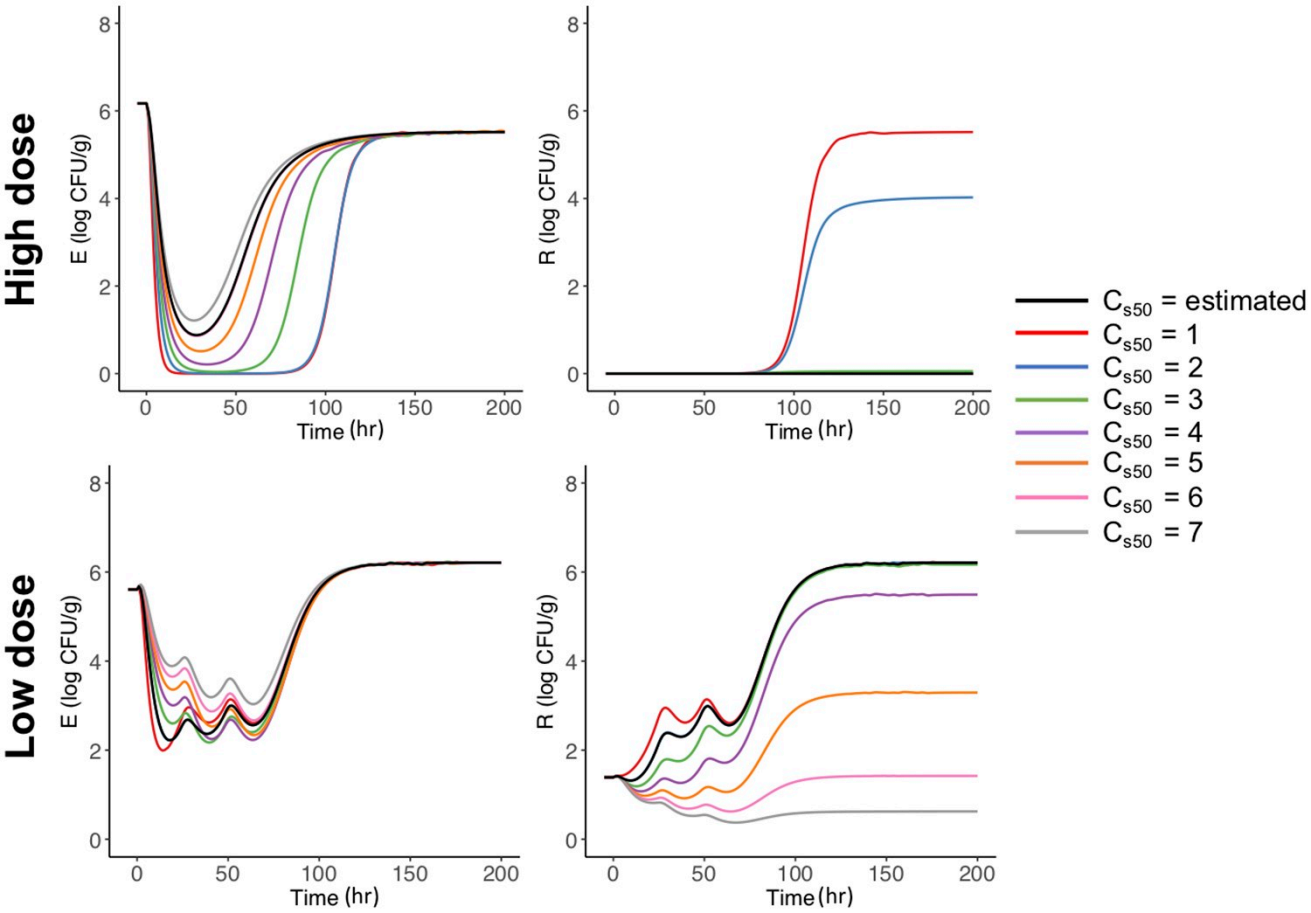
Variations in *C*_*s*50_. Simulation of variations of *C*_*s*50_ and the effects on the high (top row) and low (bottom row) dose model (1). In the simulation *C*_*s*50_ is varied from the initial estimate of 6.05 or 2.02 (black line) for the high and low dose model, respectively. We vary *C*_*s*50_ with incremental values of 7 (red line), 6 (blue line), 5 (green line), 4 (purple line), 3 (orange line), 2 (pink line) and 1 (grey line).

We begin by varying the *C*_*s*50_, 50% of sensitive bacteria killing effect. In Fig 8, we use the median parameters for the high and low dose steers in Table 1 and vary *C*_*s*50_ to see the effect it has on the amount of *E. coli* (left panel) and the total amount of *E. coli* above the ECOFF (right panel). In the high dose steers (top row) we find that when *C*_*s*50_ = 1 or 2 the high dose regimen leads to increased amounts of bacteria above the ECOFF. In the low dose steers (bottom row), we find there is always bacteria above the ECOFF, albeit for smaller *C*_*s*50_ there are more bacteria above the ECOFF. When *C*_*s*50_ < 3 in the low dose steers, we see that the final steady state of the simulation is a complete *E. coli* population above the ECOFF.

#### Variations in GIT movement

**Fig 9:**
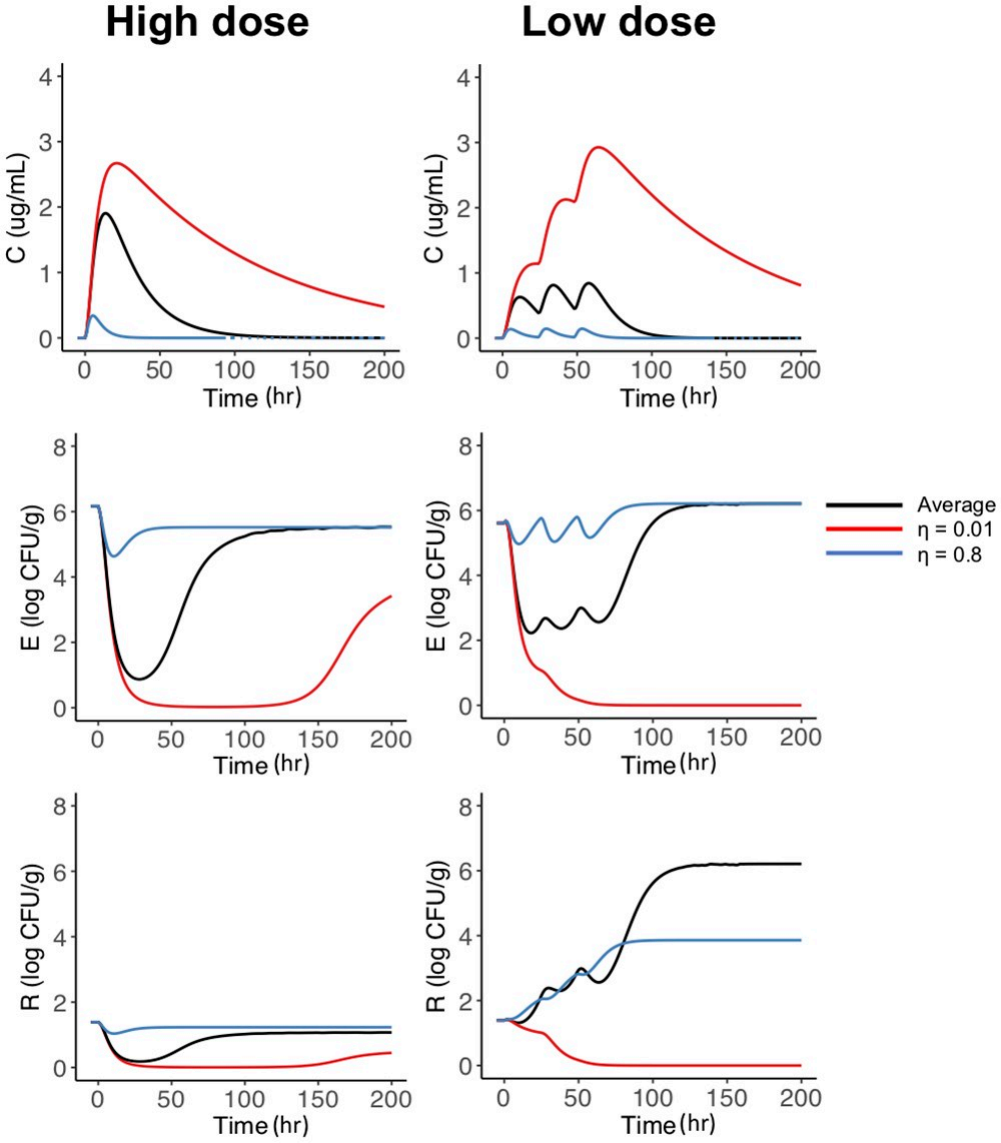
Variations in *η*. Varying *η*, the elimination of the drug from the colon, to understand the changes that occur in sick or non-normal steers in the low and high dose study. The black line represents the average steer simulation, the red line is slow movement (*η* = 0.01) and the blue line is rapid movement (*η* = 0.8).

The original cohort of steers is a healthy cohort who have similar living and feeding conditions. Using our model, we adjusted the *η* to ranges outside the predicted population distributions to simulate changes that may occur on sick animals. For example, a decreased fecal elimination rate could occur when steers stop eating during illness which leads to significantly decreased intestinal motility. An increased elimination may take place when the animal has diarrhea or increased intestinal motility. In Fig 9, we vary *η* from *η* = 0.01 (red line) to *η* = 0.8 (blue line). We increased the initial condition in the high dose simulation to *r*_0_ = 1.39 to be comparable to the low dose *r*_0_. While these variations have no effect on the plasma compartment, they do affect the colon compartment, total *E. coli*, and amount of *E. coli* above the ECOFF. In Fig 9 we see the large *η* leads to less antimicrobial drug in the colon (blue line, top row Fig 9) due to the rapid elimination, while the the small *η* leads to more antimicrobial drug in the colon (red line, top row Fig 9). The slow elimination (small *η*) leads to a decrease amount of overall *E. coli* above the ECOFF in both the high and low dosing regimens (red line, bottom row Fig 9). This is expected because of the large amounts of antibiotics in the colon, which decreases the total amount of *E. coli* and thus the subset of *E.coli* above ECOFF. Surprisingly, increasing the *η* causes analogous effects in the high and low dose treatments. In the high dose steers, increasing the *η* caused an increased amount of bacteria above the ECOFF (bottom left panel, blue line Fig 9), albeit the difference are negligible. In the low dose steers, decreasing the *η* causes less antimicrobial to reach the colon, and subsequently less bacteria above the ECOFF (bottom right panel, blue line, Fig 9).

#### Variations in the initial amount of bacteria above the ECOFF

**Fig 10:**
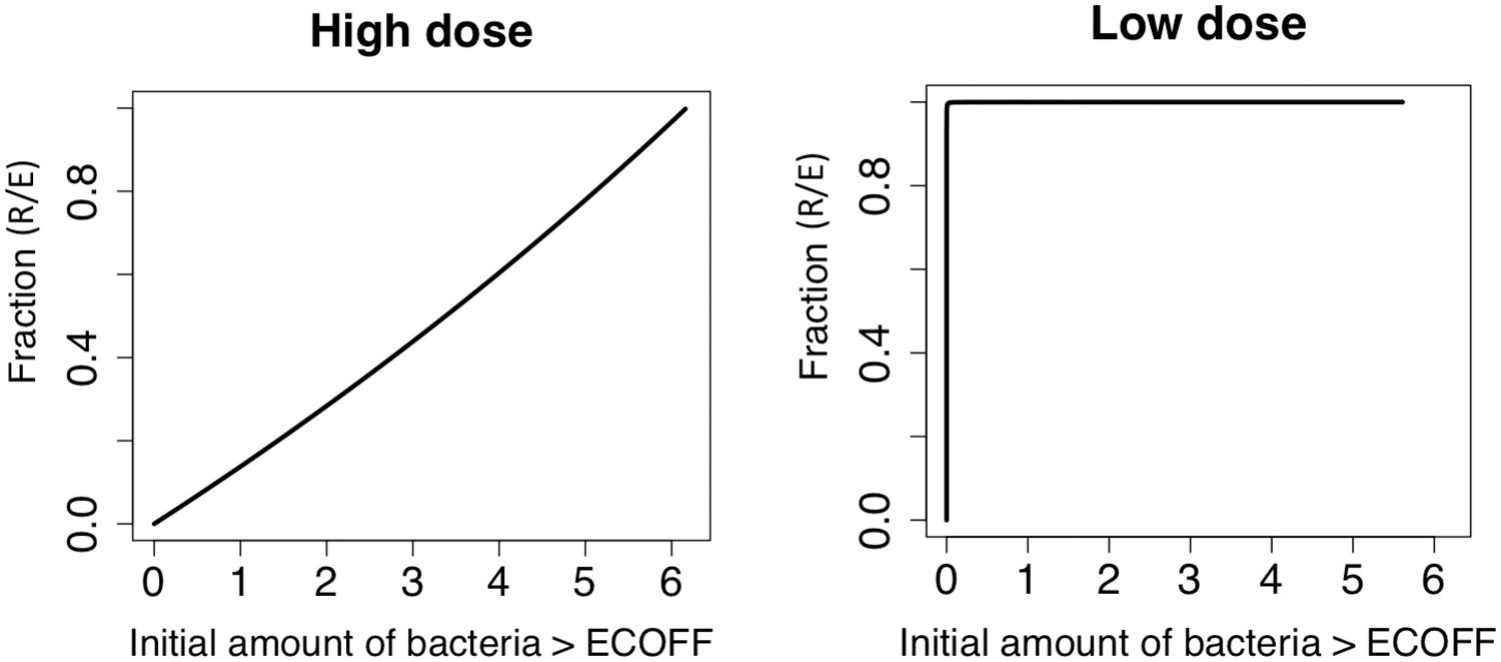
Varying initial bacteria. We shift only the initial condition *r*_0_ from 0 to the total amount of *E. coli* in each treatment and investigate how this effects the fraction of R/E, or the proportion of *E. coli* about ECOFF.

To further understand the growth of *E. coli* above the ECOFF, we simulated how changes in the initial amount of bacteria above the ECOFF affected the outcomes of the two dosing regimens. In Fig 10, left panel, we used the population parameters from the high dose steers, which had previously only predicted a completely sensitive population after treatment. We varied only the initial amount of bacteria above the ECOFF and show that as we increase the initial amount of bacteria above the ECOFF, the overall fraction of bacteria above the ECOFF increased at the end of the simulation. Similarly, in the right panel, we found that by increasing the initial amount of bacteria above ECOFF the amount of overall fraction of bacteria above the ECOFF rapidly increased. Overall, we found that by changing the initial amount of bacteria above the ECOFF alone, the steer returns to a state of primarily bacteria above the ECOFF after treatment. These results indicate that the initial amount of bacteria above the ECOFF is a strong predictor for the growth of bacteria above the ECOFF, particularly in the high dose treatment.

### Simulating decreased fitness in bacteria above the epidemiological cut-off

**Fig 11:**
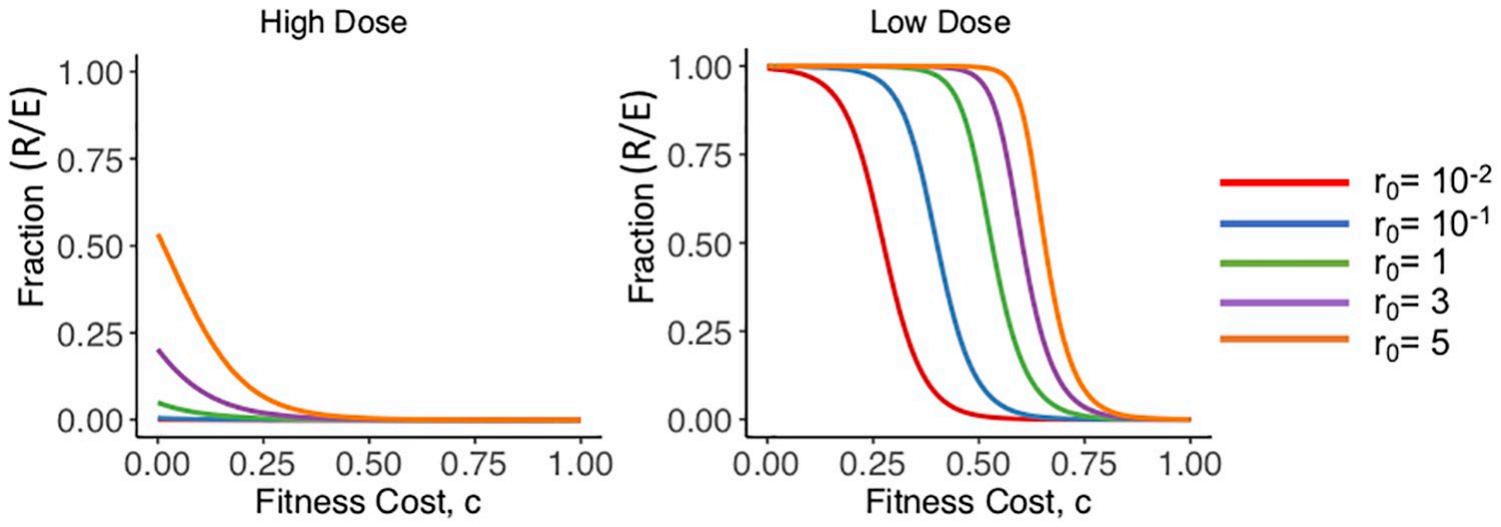
Varying fitness cost. Varying *c*, the fitness cost, to understand the effect on the ratio of *R*/*E* at 200 hours for each dosing regimen. Each line represents a different amounts of *r*_0_.

Our current model does not consider fitness cost in the growth of *E. coli* above the ECOFF. Using the previous model (1), we adjusted the differential equations to consider the effect of the fitness cost on the percentage of bacteria above the ECOFF [27, 28]. Using the estimated population parameters we re-simulated our model using the system (2), where *c* is the fitness cost. We simulated the effect of adjusting *c* from 0 to 1 on the fraction of bacteria above the ECOFF 200 hours after the beginning of the treatments. For both dosing regimens, we used the same variations of initial conditions for the initial amount of resistance bacteria i.e. *r*_0_ = 10^−2^, 10^−1^, 1, 3, and 5. In Fig 11, we plotted the trade off between *c*, x-axis, and *R*/*E*, y-axis. When the initial condition is small (*r*_0_ = 10^−2^), for all fitness costs the high dose steers also go to *R*/*E* ≈ 0. In the low dose steers with a small initial condition, we found that when *c* > 0.274, the *R*/*E* ratio is at halfway, i.e. *R*/*E* = 0.5. When the initial condition is large (*r*_0_ = 5), the high dose steers reach the halfway ratio when *c* > 0.12. In the low dose steers with the high initial condition, we find that when *c* > 0.656, the *R*/*E* ratio is at halfway.

Previous studies have shown that fitness cost can have a wide range of effects on bacteria [20]. It is thought that a single mutation can have no effect, or a fitness value as much as 0.6. Depending on the cost of each mutation, the fitness value can compound to be as much as 0.68 [20]. While our extreme selection of *c* = 1 is unlikely, it is possible that for any initial condition, the population could have an *R*/*E* ratio<0.50 depending on the fitness costs of mutations.

## Discussion

Understanding how antimicrobial drug regimens influence enteric bacteria in food animals is a relevant public health concern as food animals are a reservoir for zoonotic enteric bacteria, including resistant bacteria. In recent years, mathematical models of different complexities have been developed to investigate the underlying relationships between antimicrobial use and gut bacteria dynamics [28–31]. Most studies combined data from the literature to parameterize the models. As a result, predictions may not be accurate when compared with *in vivo* data [14]. In addition, the individual- and population-level variation and uncertainty associated with the outcomes cannot be assessed. To the best of our knowledge, our study is the first to fit a compartmental model for the dynamics of the antimicrobial drug and enteric bacteria to experimental data for assessing the collateral effects of parental treatments on gut bacteria. In addition, by using mixed-effect modeling tools, we were able to estimate individual and population-level parameters, and characterize the variation around the parameters and model predictions.

Overall, our parameters aligned with previously reported values. Our estimates of drug elimination are comparable to our group’s original fluoroquinolones study [14]. Our estimated bacterial parameters are also similar to previously used bacterial parameters in other studies. The growth rate for commensal *E.coli* previously used on a growth model was 0.17 [28], which is similar to both our high and low dose estimates. Additionally, *N*_*max*_ values have been reported between 5.5 [32] and 6.5 [33]; both of our estimations fall with in this range. While these values are only estimated for the steers in our cohort, we believe the usefulness of our study is broad because steers are the primary animal recipients of enrofloxacin.

Our model captures changes on bacteria populations mediated by competitive release and selection during treatment [34]. The model does not address emergence of *de novo* mutations or the long term replacement of bacteria after treatment. Competition among bacteria is captured by the shared carrying capacity (*N*_*max*_) and the differential selection during treatment is mediated by the parameters *C*_*s*50_ and *C*_*r*50_. The sensitivity analysis indicated that for both dose regimens, the accumulated *E. coli* population above the ECOFF was more sensitive to the killing effects (*C*_*s*50_ and *C*_*r*50_) than *N*_*max*_, which vary less in both treatments (Table 1). The model outcomes were also very sensitive to the initial proportion of *E. coli* above the ECOFF. In the high dose regimen, bacteria above the ECOFF were not amplified during treatment (Fig 2). Because the initial proportion of bacteria above the ECOFF was low, we explored the model outcome under different initial conditions beyond the ones observed in the study (Fig 10). For the high dose treatment, higher numbers of bacteria above the ECOFF at the beginning of the treatment translated into an increase in the proportion of bacteria above the ECOFF, although the increase was not proportional to the initial bacteria levels. On the other hand, for the low dose regimen, the proportion of bacteria above the ECOFF were clearly amplified independently of the initial amount (Fig 10). Overall, these findings suggest that dose regimens and initial bacteria composition are important indicators of resistance selection.

While there was extensive experimental data reported in the original study regarding the amount of antimicrobial drugs in the GIT, the timing of sampling was inconsistent across the dosing studies and cohorts. Moreover, there was large variation in the initial amount of bacteria above ECOFF between the single dose and multiple dose steers. These variations across cohorts do effect our parameter estimates in the current study, but there is enough data to highlight the dynamics within the cohorts during the course of the previous study. A more significant limitation of the original study was lack of MICs reported when identifying the amount of bacteria above the ECOFF. For some steers MICs were only collected at the beginning and end of the study, leaving little understanding of the changes in bacteria during dosing. Additionally, there were only 8 isolates collected leaving large room for error when estimating the bacteria population above the ECOFF. We believe the derivation of our current model—using a total population and a population above the ECOFF instead of above and below the ECOFF population—helps to overcome the limited isolates. Additionally, our current results highlight that the speed of movement through the colon may be able to mitigate the growth of bacteria above the ECOFF. Slow colon movement reduced growth of all bacteria in both dosing regimens, while rapid colon movement reduced growth of *E. coli* above the ECOFF in the low dosing regimens (Fig 9). This indicates that changes in motility in sick animals may also influence resistance selection during treatment.

Despite the limitations, our model provides a basic framework to model the effects of antimicrobial drugs on enteric bacteria. Previous work [35] highlighted the need for a such a model and suggests this type of model could guide future study design. Additionally, the unique GIT dataset utilized herein is a key factor in calibrating the model to estimate data-driven parameters. Previous work created a similar model to measure withdrawal times from feedlot to slaughterhouse in steers with limited data [31] and found similar results regarding the importance of the colon movement, despite the limited data. Our work could be easily adjusted for other data sets, of this drug or other antimicrobial drugs which we believe would further validate the model and impact future studies. Additionally, this work could by adapted to study withdrawal times of antimicrobial drugs and their effect on antimicrobial resistance.

In conclusion, this model is the first attempt to combine the concentrations of antimicrobial drugs with their effect on the bacteria concentrations with in the GIT. Our results highlight the need for further data collection to gain a deeper understanding of such dynamics. Our results also suggest that the initial susceptibility of *E. coli* in the GIT is a strong indicator of how steers will respond to antimicrobial drug treatment. This model highlights the importance of the judicious use of antimicrobial drugs and need for isolate testing prior to initiating an antimicrobial drug treatment regimen.

## Supporting information

S1 File Supporting file with code and parameter estimations. (CSV)

S2 File Data used to perform numerical simulations. (CSV)

S1 Appendix COMBOS. (PDF)

S2 Appendix Monolix code. The monolix code used to run the low dose simulations. The highdose code files are comparable with minor adjustments. The data is in the paper from Foster et al., 2015.
(PDF)

S1 Table Parameter summary of high dose steers. (PDF)

S2 Table Parameter summary of low dose steers. (PDF)

## References

[1] Riviere J, Papich M. Veterinary pharmacology and therapeutics. John Wiley & Sons; 2018.

[2] Collignon P, Conly J, Andremont A, McEwen S, et al World Health Organization ranking of antimicrobials according to their importance in human medicine: a critical step for developing risk management strategies to control antimicrobial resistance from food animal production. Clin Infect Dis. 2016;63:1087–93. 10.1093/cid/ciw475 27439526

[3] Shryock T, Richwine A. The interface between veterinary and human antibiotic use. Ann N Y Acad Sci. 2010;1213:92–105. 10.1111/j.1749-6632.2010.05788.x 20946576

[4] Ternhag A, Asikainen T, Giesecke J, Ekdahl K. A meta-analysis on the effects of antibiotic treatment on duration of symptoms caused by infection with Campylobacter species. Clin Infect Dis. 2007;44:696–700. 10.1086/509924 17278062

[5] Kariuki S, Gordon M, Feasey N, Parry C. Antimicrobial resistance and management of invasive Salmonella disease. Vaccine. 2015;33:C21–9. 10.1016/j.vaccine.2015.03.102 25912288 PMC4469558

[6] Doyle M. Multidrug-resistant pathogens in the food supply. Foodborne pathog dis. 2015;12:261–79. 10.1089/fpd.2014.1865 25621383

[7] Giguère S, Prescott J, Dowling P. Antimicrobial therapy in veterinary medicine. John Wiley & Sons; 2013.

[8] Alvarez A, Pérez M, Prieto J, Molina A, Real R, Merino G. Fluoroquinolone efflux mediated by ABC transporters. J Pharm Sci. 2008;97:3483–93. 10.1002/jps.21233 18200507

[9] Donskey CJ, Helfand MS, Pultz NJ, Rice LB. Effect of parenteral fluoroquinolone administration on persistence of vancomycin-resistant Enterococcus faecium in the mouse gastrointestinal tract. Antimicrob Agents Chemother. 2004;48:326–8. 10.1128/AAC.48.1.326-328.2004 14693559 PMC310198

[10] Redgrave L, Sutton S, Webber M, Piddock L. Fluoroquinolone resistance: mechanisms, impact on bacteria, and role in evolutionary success. Trends microbiol.2014; 22:438–45. 10.1016/j.tim.2014.04.007 24842194

[11] Wiuff C, Lykkesfeldt J, Svendsen O, Aarestrup F. The effects of oral and intramuscular administration and dose escalation of enrofloxacin on the selection of quinolone resistance among Salmonella and coliforms in pigs. Res Vet Sci. 2003;75:185–93. 10.1016/s0034-5288(03)00112-7 13129666

[12] Chantziaras I, Smet A, Haesebrouck F, Boyen F, Dewulf J. Studying the effect of administration route and treatment dose on the selection of enrofloxacin resistance in commensal Escherichia coli in broilers. J Antimicrob Chemother. 2017;72:1991–2001. 10.1093/jac/dkx104 28419236

[13] McDermott P, Bodeis S, English L, White D, et al Ciprofloxacin resistance in Campylobacter jejuni evolves rapidly in chickens treated with fluoroquinolones. J Infect Dis. 2002; p. 837–40. 10.1086/339195 11920303

[14] Foster D, Jacob M, Warren C, Papich M. Pharmacokinetics of enrofloxacin and ceftiofur in plasma, interstitial fluid, and gastrointestinal tract of calves after subcutaneous injection, and bactericidal impacts on representative enteric bacteria. J Vet Pharmacol Ther. 2015;39:62–71. 10.1111/jvp.12236 25989138

[15] Ferguson K, Jacob M, Theriot C, Callahan B, et al Dosing Regimen of Enrofloxacin Impacts Intestinal Pharmacokinetics and the Fecal Microbiota in Steers. Front Microbiol. 2018;9:2190 10.3389/fmicb.2018.02190 30283418 PMC6156522

[16] Kaartinen L, Salonen M, Älli L, Pyörälä S. Pharmacokinetics of enrofloxacin after single intravenous, intramuscular and subcutaneous injections in lactating cows. J Vet Pharmacol Ther. 1995;18:357–62. 10.1111/j.1365-2885.1995.tb00604.x 8587154

[17] Clinical, Institute LS. Performance Standards for Antimicrobial Disk and Dilution Susceptibility Tests for Bacteria Isolated From Animals; 2018.

[18] ESCMID. EUCAST; 2018. http://www.eucast.org.

[19] Grobbel M, Lübke-Becker A, Wieler L, Froyman R, Friederichs S, Filios S. Comparative quantification of the in vitro activity of veterinary fluoroquinolones. Vet microbiol. 2007;124:73–81. 10.1016/j.vetmic.2007.03.017 17498893

[20] Marcusson L, Frimodt-Møller N, Hughes D. Interplay in the selection of fluoroquinolone resistance and bacterial fitness. PLoS pathog. 2009;5:e1000541 10.1371/journal.ppat.1000541 19662169 PMC2714960

[21] Meshkat N, Kuo C, DiStefano J III. On finding and using identifiable parameter combinations in nonlinear dynamic systems biology models and COMBOS: A novel web implementation. PloS One. 2014;9:e110261 10.1371/journal.pone.0110261 25350289 PMC4211654

[22] Delyon B, Lavielle M, Moulines E. Convergence of a stochastic approximation version of the EM algorithm. Ann Stat. 1999;27:94–128.

[23] Massot M., Couffignal C., Clermont O., D’Humières C., Chatel J., Plault N., et al Day-to-day dynamics of commensal Escherichia coli in Zimbabwean cows evidence temporal fluctuations within a host-specific population structure Appl Environ Microbiol. 2017; 83:e00659–17. 10.1128/AEM.00659-17 28411228 PMC5478991

[24] Mentré F, Gomeni R. A two-step iterative algorithm for estimation in nonlinear mixed-effect models with an evaluation in population pharmacokinetics. J Biopharm Stat. 1995; p. 141–58. 10.1080/10543409508835104 7581424

[25] Bertrand I, Alexandre J, Gilles P, et al. Sensitivity: Global Sensitivity Analysis of Model Outputs. 2018;.

[26] Saltelli A, Tarantola S, Chan K. A quantitative model-independent method for global sensitivity analysis of model output. Technometrics. 1999;41:39–56. 10.1080/00401706.1999.10485594

[27] Ternent L, Dyson R, Krachler A, Jabbari S. Bacterial fitness shapes the population dynamics of antibiotic-resistant and-susceptible bacteria in a model of combined antibiotic and anti-virulence treatment. J Theo Biol. 2015;372:1–11. 10.1016/j.jtbi.2015.02.011 PMC4396697 25701634

[28] Volkova V, Lanzas C, Lu Z, Gröhn Y. Mathematical model of plasmid-mediated resistance to ceftiofur in commensal enteric Escherichia coli of cattle. PLoS One. 2012;7:e36738 10.1371/journal.pone.0036738 22615803 PMC3353932

[29] Lanzas C, Lu Z, Grohn Y. Mathematical modeling of the transmission and control of foodborne pathogens and antimicrobial resistance at preharvest. Foodborne Pathog Dis. 2011;8:1–10. 10.1089/fpd.2010.0643 21043837 PMC3123936

[30] Ahmad A, Græsbøll K, Christiansen L, Toft N, Matthews L, Nielsen S. Pharmacokinetic-pharmacodynamic model to evaluate intramuscular tetracycline treatment protocols to prevent antimicrobial resistance in pigs. Antimicrob Agents Chemother. 2015;59:1634–42. 10.1128/AAC.03919-14 25547361 PMC4325798

[31] Cazer C, Ducrot L, Volkova V, Gröhn Y. Monte Carlo simulations suggest current chlortetracycline drug-residue based withdrawal periods would not control antimicrobial resistance dissemination from feedlot to slaughterhouse. Front Microbiol. 2017;8 10.3389/fmicb.2017.01753 29033901 PMC5627025

[32] Aslam M, Greer G, Nattress F, Gill C, McMullen L. Genetic diversity of Escherichia coli recovered from the oral cavity of beef cattle and their relatedness to faecal *E. coli*. Lett Appl Microbiol. 2004;39:523–7. 10.1111/j.1472-765X.2004.01620.x 15548305

[33] Daniels J, Call D, Hancock D, Sischo W, Baker K, Besser T. Role of ceftiofur in selection and dissemination of blaCMY-2-mediated cephalosporin resistance in Salmonella enterica and commensal Escherichia coli isolates from cattle. Appl Environ Microbiol. 2009;75:3648–55. 10.1128/AEM.02435-08 19376926 PMC2687309

[34] Day T, Huijben S, Read A. Is selection relevant in the evolutionary emergence of drug resistance? Trends in Microbiol. 2015;23:126–33. 10.1016/j.tim.2015.01.005 PMC4494118 25680587

[35] Volkova V, KuKanich B, Riviere J. Exploring post-treatment reversion of antimicrobial resistance in enteric bacteria of food animals as a resistance mitigation strategy. Foodborne path dis. 2016;13:610–7. 10.1089/fpd.2016.2152 27552491

