# Supplementary material for: The effect of enrofloxacin on enteric Escherichia coli: Fitting a mathematical model to in vivo data
Source: PLoS One. 2020 Jan 31;15(1):e0228138. doi: 10.1371/journal.pone.0228138 (PMC6993981; doi:10.1371/journal.pone.0228138)
Supplement: S1 Appendix — (PDF) [file pone.0228138.s003.pdf]

The effect of enrofloxacin on enteric *Escherichia coli*: fitting a mathematical model to *in vivo* data

Samantha Erwin*[1,3], Derek M. Foster[1], Megan E. Jacob[1], Mark G. Papich[2], Cristina Lanzas[1],

1 Department of Population Health and Pathobiology, College of Veterinary Medicine, North Carolina State University, Raleigh, NC, United States of America
2 Department of Molecular and Biomedical Sciences, College of Veterinary Medicine, North Carolina State University, Raleigh, NC, United States of America
3 Biomedical Sciences, Engineering, and Computing Group, Oak Ridge National Laboratory, Oak Ridge, TN, United States of America

*erwinsh@ornl.gov

Supporting information

S1 Appendix. COMBOS. The precise code used in the web application combos is:

```
dx1/dt = k9*u1-k1*x1;
   dx2/dt = k1*x1-k2*x2;
   dx3/dt = k3*(1-x3/k4)*x3-x4*x2/(x2-k6)-(x3-x4)*x2/(x2-k5);
   dx4/dt = k3*(1-x3/k4)*x4-x4*x2/(x2-k6);
   y1 = x1; y2 = x2; y3 = x3; y4 = x4;
   x1(0) = 0;x2(0) = 0;
```
