# Supplementary material for: The effect of enrofloxacin on enteric Escherichia coli: Fitting a mathematical model to in vivo data
Source: PLoS One. 2020 Jan 31;15(1):e0228138. doi: 10.1371/journal.pone.0228138 (PMC6993981; doi:10.1371/journal.pone.0228138)
Supplement: S2 Appendix — The monolix code used to run the low dose simulations. The highdose code files are comparable with minor adjustments. The data is in the paper from Foster et al., 2015. (PDF) [file pone.0228138.s004.pdf]

S2 Appendix. Monolix Code. The monolix code used to run the low dose simulations. The highdose code files are comparable with minor adjustments. The data is in the paper from Foster et al., 2015.

Model File

```
DESCRIPTION: Low dose model file
  [LONGITUDINAL]
  input = {beta, alpha, eta, f, r, Nmax, Cs50, Cr50}
  f = {use = regressor}
  EQUATION:
  odeType = stiff
  k=0.44
  V=1
  R_0 = r
  E_0 = f
  {S} = pkmodel(V, k)
  ddt_P = beta*k*S - alpha*P
  ddt_C = alpha*P - eta*C
  ddt_E = sigma*(1 - E/Nmax)*E-(C/(C+Cs50))*(E-R)-(C/(C+Cr50))*R
  ddt_R = sigma*(1-E/Nmax)*R - (C/(C+Cr50))*R
  OUTPUT: output = {P,C,E,R}
```

Automatically generated low dose script

```
; this script is generated automatically
  <DATAFILE>
  [FILEINFO]
  file = 'LowDose_Ecoli.csv'
  delimiter = comma
  header = {ID, TIME, Y, WHAT, YTYPE, DOSE, m_f, m_r}
  [CONTENT]
  ID = {use=identifier}
  TIME = {use=time}
  Y = {use=observation, name={y1, y2, y3, y4}, ytype={1, 2, 3, 4},
  type={continuous, continuous, continuous, continuous}}
  YTYPE = {use=observationType}
  DOSE = {use=amount}
  m_f = {use = regressor}
  <MODEL>
  [INDIVIDUAL]
  input = {beta_pop, omega_beta, alpha_pop, omega_alpha, eta_pop,
  omega_eta, r_pop, omega_r, Nmax_pop, omega_Nmax, Cs50_pop, omega_Cs50,
  Cr50_pop, omega_Cr50}
  DEFINITION:
  beta = {distribution=lognormal, typical=beta_pop, sd = omega_beta}
  alpha = {distribution=lognormal, typical=alpha_pop, sd = omega_alpha}
  eta = {distribution=lognormal, typical=eta_pop, sd = omega_eta}
  r = {distribution=lognormal, typical=r_pop, sd = omega_r}
  Nmax = {distribution=lognormal, typical=Nmax_pop, sd = omega_Nmax}
  Cs50 = {distribution=lognormal, typical=Cs50_pop, sd = omega_Cs50}
  Cr50 = {distribution=lognormal, typical=Cr50_pop, sd = omega_Cr50}
  [LONGITUDINAL]
  input = {a1, a2, a3, a4}
  file = './Low_Bacteria_Model.txt'
  DEFINITION:
  y1 = {distribution=normal, prediction=P, errorModel=constant(a1)}
  y2 = {distribution=normal, prediction=C, errorModel=constant(a2)}
  y3 = {distribution=normal, prediction=E, errorModel=constant(a3)}
  y4 = {distribution=normal, prediction=R, errorModel=constant(a4)}
  <FIT>
  data = {y1, y2, y3, y4}
  model = {y1, y2, y3, y4}
  <PARAMETER>
  beta_pop = {value=0.001, method=MLE}
  omega_beta = {value=1, method=MLE}
  alpha_pop = {value=0.1, method=MLE}
  omega_alpha = {value=1, method=MLE}
  eta_pop = {value=0.09, method=MLE}
  omega_eta = {value=1, method=MLE}
  r_pop = {value=2, method=MLE}
  omega_r = {value=1, method=MLE}
  Nmax_pop = {value=5, method=MLE}
  omega_Nmax = {value=1, method=MLE}
  Cs50_pop = {value=2, method=MLE}
  omega_Cs50 = {value=1, method=MLE}
```

```
Cr50_pop = {value=6, method=MLE}
omega_Cr50 = {value=1, method=MLE}
a1 = {value=1, method=MLE}
a2 = {value=0.065998, method=MLE}
a3 = {value=0.227992, method=MLE}
a4 = {value=0.239269, method=MLE}
<MONOLIX>
[TASKS]
populationParameters()
fim(method = Linearization)
plotResult()
[SETTINGS]
GLOBAL:
exportpath = 'Lowdose_project'
nbchains = 9
POPULATION:
mixturesinitbeta = 1
```
