# Supplementary material for: The effect of enrofloxacin on enteric Escherichia coli: Fitting a mathematical model to in vivo data
Source: PLoS One. 2020 Jan 31;15(1):e0228138. doi: 10.1371/journal.pone.0228138 (PMC6993981; doi:10.1371/journal.pone.0228138)
Supplement: S1 Table — (PDF) [file pone.0228138.s005.pdf]

Tables. **Parameter Estimates.** In Tables 1 and Tables 2 we summarize the parameters estimates for each of the individual steers.

Table 1. Parameter summary of high dose steers

Parameters	min	Q1	median	Q3	max
β	0.000705	0.00114	0.00137	0.00165	0.00177
α	0.0895	0.148	0.186	0.195	0.216
η	0.0232	0.0308	0.0457	0.099	0.102
σ	0.145	0.183	0.218	0.251	0.321
N_{max}	5.35	5.38	5.53	5.66	5.72
C_{s50}	3.95	4.06	6.05	6.81	15.3
r_0	0.00272	0.00394	0.00979	0.0169	4.37

Summary of the parameter estimates of all six steers in the high dose simulation.
