# Supplementary material for: The effect of enrofloxacin on enteric Escherichia coli: Fitting a mathematical model to in vivo data
Source: PLoS One. 2020 Jan 31;15(1):e0228138. doi: 10.1371/journal.pone.0228138 (PMC6993981; doi:10.1371/journal.pone.0228138)
Supplement: S2 Table — (PDF) [file pone.0228138.s006.pdf]

Table 2. Parameter summary of low dose steers

Parameters	min	Q1	median	Q3	max
β	0.000995	0.00113	0.00166	0.0019	0.00265
α	0.111	0.134	0.15	0.163	0.189
η	0.085	0.0854	0.0855	0.0857	0.0857
σ	0.187	0.188	0.188	0.189	0.19
N_{max}	5.68	5.82	6.22	6.48	6.96
C_{s50}	1.12	1.48	1.67	4.32	4.68
C_{s50}	3.93	4.53	6.04	7.93	9.08
r_0	0.554	1.18	1.66	2.16	3.14

Summary of the parameter estimates of all six steers in the low dose simulation.
